# FALL RISK BEHAVIORS AND INTRINSIC RISK FACTORS FOR FALLS IN INDIGENOUS AND NON-INDIGENOUS RURAL OLDER ADULTS

**DOI:** 10.1093/geroni/igz038.1758

**Published:** 2019-11-08

**Authors:** Andre G Bouweraerts, Justus Ortega

**Affiliations:** Humboldt State University, Arcata, California, United States

## Abstract

Within California, older adults living in rural counties have reported higher rates of falls than urban dwelling older adults. Although many Indigenous people live in rural areas, it is unclear whether the rate of falls among Indigenous older adults is similar to that of non-indigenous older adults living in rural areas. Thus, the purpose of this study was to examine fall risk behaviors and intrinsic risk factors for falls in rural dwelling Indigenous (N = 89), and non-Indigenous (N = 68) older adults 60-95 years of age living in California. Results showed that both Indigenous and non-Indigenous older adults share similarly high fall rates, but there are a much greater number of Indigenous older adults falling multiple times a year. Moreover, fall risk behaviors and intrinsic fall risk factors were significantly different between Indigenous and non-Indigenous rural-dwelling older adults. Future studies should investigate falls and fall risk factors in different tribes/locations of Indigenous older adults to better understand whether these risk factors differ among tribes. Moreover, it would be beneficial for future studies to assess the effectiveness of fall prevention exercises on fall risk in these communities. Information gained from this study helps to inform clinicians and researchers alike about the prevalence of falls and factors contributing to falls among Indigenous older adults living in rural communities; and helps to influence decisions in the future of programs for reducing fall risk in this often neglected population.

